# Association between body mass index and cognitive impairment in Chinese older adults

**DOI:** 10.3389/fpubh.2023.1255101

**Published:** 2023-10-18

**Authors:** Wenshuo Dong, Lichao Kan, Xinyue Zhang, Mengli Li, Meijuan Wang, Yingjuan Cao

**Affiliations:** ^1^School of Nursing and Rehabilitation, Shandong University, Jinan, Shandong, China; ^2^Department of Nursing, Qilu Hospital, Cheeloo College of Medicine, Shandong University, Jinan, Shandong, China; ^3^Nursing Theory and Practice Innovation Research Center, Shandong University, Jinan, Shandong, China

**Keywords:** older adults, BMI, cognitive impairment, restricted cubic splines, does-response

## Abstract

**Background:**

The association between body mass index (BMI) and the risk of cognitive impairment remains uncertain. Relatively few studies have analyzed the dose–response relationship between BMI and cognitive impairment. This article utilized nationally representative longitudinal data to assess the association between BMI and cognitive impairment in Chinese older adults.

**Objective:**

The present study aimed to analyze the association between BMI and cognitive impairment in Chinese older people, including an investigation of gender differences and the dose–response relationship.

**Methods:**

Data were obtained from the China Health and Retirement Longitudinal Study database in 2015 and 2018. The present study used logistic regression to analyze the relationship between baseline BMI and cognitive impairment, and adopted a restricted cubic spline model to plot dose–response curves for baseline BMI and prevalence of risk of cognitive impairment.

**Results:**

The mean BMI of the survey population was 23.48 ± 3.66 kg/m^2^, and the detection rate of cognitive impairment was 34.2%. Compared to the normal weight group (18.5 ≤ BMI < 23.9 kg/m^2^), the odds ratio (OR) for cognitive impairment was 1.473 (95% CI: 1.189–1.823) in the underweight group (BMI < 18.5 kg/m^2^), whereas the corresponding OR was 0.874 (95% CI: 0.776–0.985) for the overweight or obese group (BMI ≥ 24.0 kg/m^2^) after adjusting for confounders. Gender subgroup analysis showed that overweight or obese older women were less likely to develop cognitive impairment (OR = 0.843; 95% CI: 0.720–0.987). The results of the restricted cubic spline analysis revealed a curvilinear L-shaped relationship between BMI and the risk of cognitive impairment (*P* non-linearity <0.05). In particular, the risk of cognitive impairment was higher at a lower baseline BMI. In contrast, BMI in the range of 23.2–27.8 kg/m^2^ was associated with a decreased risk of cognitive impairment.

**Conclusion:**

BMI is a dose-dependent related factor for cognitive impairment in Chinese older adults. Being underweight is a risk factor for the development of cognitive impairment, while being overweight or obese is less likely to have cognitive impairment, particularly in female older people. Keeping BMI ranging from 23.2–27.8 kg/m^2^ in older adults can help maintain cognitive function.

## 1. Introduction

Population aging is a significant global public health issue, but it is particularly prominent in China. According to the Seventh National Census of China, the country has 264 million people over the age of 60, which represents 18.7% of the population (1). This percentage exceeds the global average value. As the progress of global aging intensifies, research focused on cognitive impairment is being increasingly conducted. This is because cognitive impairment is closely linked to the onset of dementia, which is a common chronic disease among older adults. In a cross-sectional study published in *Lancet*, the prevalence of mild cognitive impairment among Chinese adults aged 60 years and above was 15.5%, accounting for approximately 38 million cases (2). Given the high prevalence in older adults, cognitive impairment leads to substantial nursing costs, thereby negatively impacting the living quality of older people and generating a huge burden on caregivers and society as a whole (3–5). Despite this preoccupation, there are currently no effective treatments for dementia. Therefore, identifying potential dangerous factors for cognitive impairment, in particular modifiable dangerous factors, is crucial for preventing or delaying the progression of dementia in older adults.

Underweight, overweight, and obesity are correlated with risk of disability risk and all-cause mortality (6), which are usually categorized by body mass index (BMI). However, the relationship between these BMI categories and the risk of cognitive impairment remains uncertain (7, 8). Previous studies have shown that being underweight was a strong risk factor for the onset of cognitive impairment (9, 10). Recent studies involving older populations have shown that being overweight or obese may have a positive effect on cognition (11, 12). Nevertheless, recent research on older individuals in Colombia has revealed that being overweight or obese is not associated with cognitive decline progression (13). In addition, several researches have reported gender differences in the relationship between BMI and cognitive impairment. Chen et al. showed an increased incidence of mild cognitive impairment in older women with low BMI and older men with high BMI (14). While a large Korean cohort study found that older women who were overweight or obese had a significantly lower risk of cognitive impairment, there was no such association in men (15). Up until now, gender differences in different types of BMI and the development of cognitive impairment are still inadequate. Although several researches have indicated an association between BMI and cognitive impairment. However, considering that the dependent and independent variables may not satisfy a linear relationship, most of them were mainly limited to classifying BMI as a categorical variable (underweight, normal, overweight or obese) to be included in the analysis model. Therefore, it was not possible to observe how the risk of cognitive impairment changed with subtle changes in BMI, and it was not possible to show a dose–response relationship between them.

In China, most studies on the relationship between BMI and cognitive function have used a cross-sectional design and conducted within a specific region, with controversial findings (14, 16). Therefore, our present study aimed to evaluate the association between different baseline BMI categories and cognitive impairment in the aging Chinese population, including an investigation of gender differences and the dose–response relationship between them based on data from the China Health and Retirement Longitudinal Study (CHARLS) database.

## 2. Methods

### 2.1. Data and study sample

**Figure 1:**
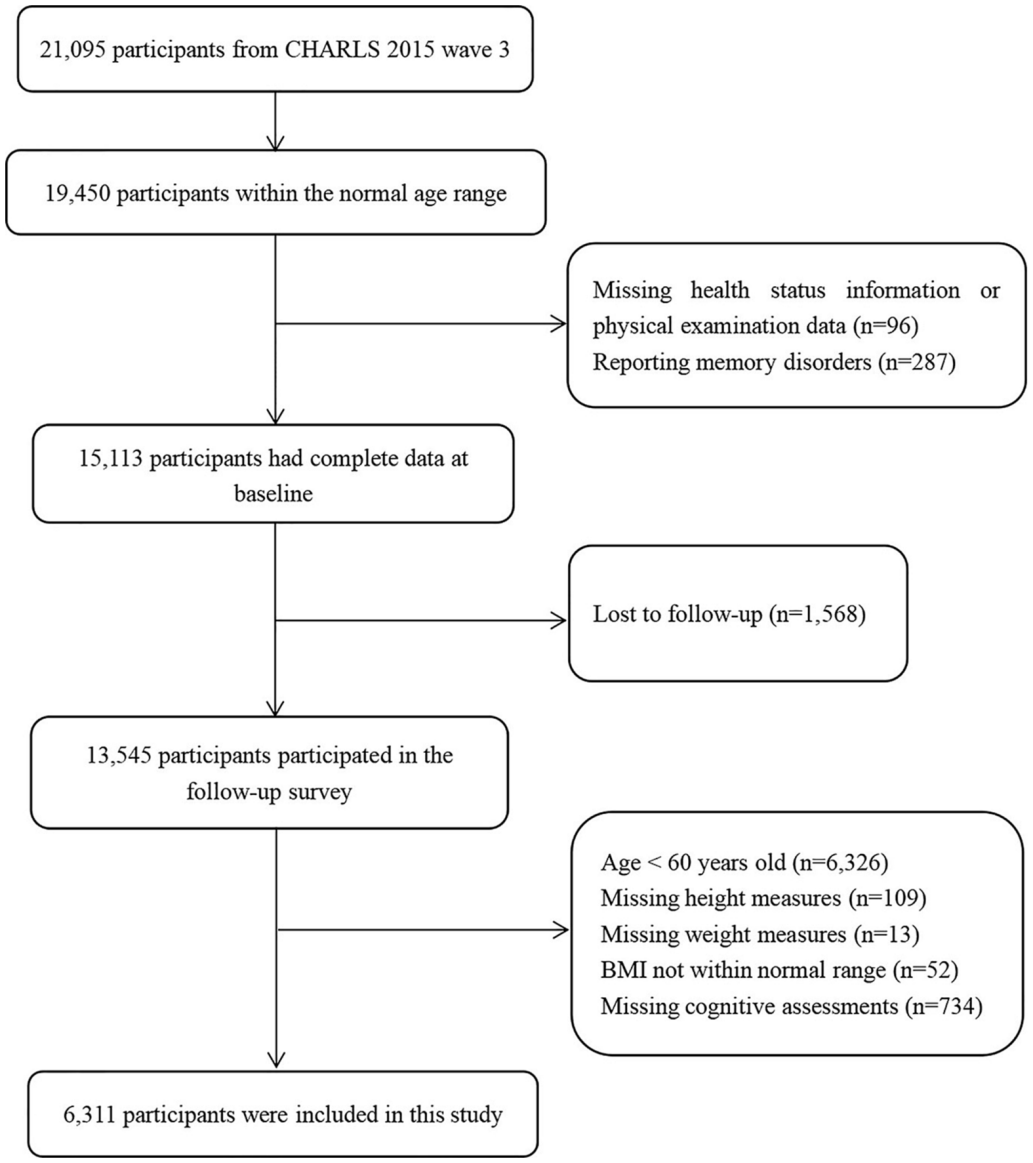
Flowchart of study participant selection.

The CHARLS is an ongoing national longitudinal survey that includes a large nationally representative sample of Chinese adults aged 45 years and older, along with their spouses. The national baseline survey was initiated in 2011 and included 17,708 respondents, with follow-up surveys every 2 years thereafter (17). The survey contained questions regarding basic personal information, family structure, and health status (18). In the present study, we used data from two waves of the CHARLS conducted in 2015 and 2018. The 2015 sample was used as the baseline sample and included 21,095 respondents. We excluded respondents who did not complete follow-up, those with missing height and weight measurements at baseline, and those with missing values on cognitive tests at follow-up; the final sample size was 6,311 respondents. Figure 1 shows the flowchart of participant selection.

### 2.2. Measures

#### 2.2.1. BMI

BMI was calculated from weight and height values (weight in kg divided by height squared in cm) measured at baseline. To determine the BMI category at baseline, the BMI value was classified into the following three groups according to the recommended Chinese guidelines (19): underweight (BMI < 18.5 kg/m^2^), normal weight (18.5 ≤ BMI < 23.9 kg/m^2^), and overweight or obese (BMI ≥ 24.0 kg/m^2^). BMI values outside the biologically plausible range of 15 to 50 kg/m^2^ were excluded from the analysis (20).

#### 2.2.2. Cognitive assessment

In the present study, cognitive function was measured using the Mini-Mental State Examination (MMSE), a scale developed by Folstein et al. (21). This scale assesses several cognitive domains such as attention and calculation, verbal ability, memory, orientation, and delayed memory. The scores of the cognitive assessment scale range from 0 to 30, with higher scores indicating superior cognition. It should be noted that the educational level of the evaluated individuals considerably impacts the outcomes of the MMSE (22). In the present study, individuals were considered to have cognitive impairment if their score was ≤17 for illiteracy, ≤ 20 for those with primary school education, and ≤ 24 for those with a junior high school degree or higher (23, 24). Cronbach’s α of the MMSE was 0.895 in this study.

#### 2.2.3. Covariates

In the present study, confounding factors such as demographic characteristics, socioeconomic status, health status, and social participation were considered as covariates. Demographic characteristics included age (60–64, 65–69, 70–74, or ≥ 75 years), gender (male or female), marital status (married, unmarried, divorced, or widowed), and place of residence (urban or rural). Socioeconomic status included educational level (illiterate, primary, or junior high school and above), average household income (low or high), and occupation (unemployed, managers and professionals, or agriculture and manual workers). The average household *per capita* income of this sample (11445.76 Yuan) was used as the cut-off point to classify low or high income. Health status included smoking (current, ever, or never), alcohol consumption (more than once a month, less than once a month, or never), vision status, hearing status, nighttime sleep duration, activities of daily living ability, depressive symptoms, and chronic disease comorbidity status. The activities of daily living score comprises two components: the Physical Self-Maintenance Scale (PSMS) and the Instrumental Activities of Daily Living (IADL) (25). The PSMS examines six distinct activities, including eating, dressing, bathing, getting in and out of bed, toileting, and controlling bowel movements, while the IADL covers six different activities, including cooking, doing housework, shopping, taking medication, managing finances, and making phone calls. If all 12 activities were performed without difficulty by the older participants, their ability to perform daily living activities was considered not impaired; otherwise, it was considered impaired. The 10-item Center for Epidemiologic Studies Depression Scale (CESD-10) short form was used to assess depressive symptoms of the participants. Each item was scored on a 4-point scale, and the total score ranged from 0 to 30, with a score ≥ 10 indicating the presence of depressive symptoms (26). Nighttime sleep duration was assessed by self-report at baseline. The presence of comorbidities and chronic conditions was measured through self-reported chronic conditions, including hypertension, stroke, asthma, heart disease, diabetes, lung disease, kidney disease, and so on. Lastly, social participation status was evaluated based on the number of social activities attended by the participants in the past month.

### 2.3. Statistical analysis

Statistical analysis was conducted using SPSS 26.0 and Stata 16.0 software, while R software version 4.2.2 was used for generating graphs. Categorical variables were expressed as frequency (N) and percentage (%). The ***χ***^***2***^ test was used for comparisons between groups. This study adopted a binary logistic regression model to determine the association between BMI [underweight (BMI < 18.5 kg/m^2^) and overweight or obese (BMI ≥ 24.0 kg/m^2^)] and cognitive impairment by using normal weight (18.5 ≤ BMI < 23.9 kg/m^2^) as a reference. Three models were used for logistic regression analysis: model 1, which was not adjusted for confounders; model 2, which was adjusted for age and gender; and model 3 was further adjusted for marital status, area of residence, education, average household income, occupation, smoking status, alcohol consumption, hearing status, ability to perform ADL, nighttime sleep duration, depressive symptoms, and social participation status. Odds ratios (ORs) and 95% confidence intervals (CI) were calculated. A dose–response relationship curve between BMI, which was considered a continuous variable, and the risk of developing cognitive impairment was fitted using restricted cubic splines. The analysis code can be found in the Supplementary material. A two-tailed *p*-value of <0.05 was considered statistically significant.

## 3. Results

### 3.1. Baseline characteristics of the study participants

This study included 6,311 older adults (3,072 males and 3,239 females) aged between 60 to 93 years, with a median age of 66 years. Of the total participants, 4,802 (76.1%) were from rural areas, 3,570 (56.6%) were illiterate, and 1,163 (18.4%) were married. The mean BMI of the study participants was 23.48 (SD = 3.66) kg/m^2^. Among the study population, 440 (7.0%) individuals were underweight, 3,266 (51.8%) individuals had normal weight, and 2,605 (41.2%) individuals were overweight or obese. The baseline characteristics of the participants are presented in Table 1.

**Table 1:** Baseline characteristics of the study participants.

	Total	Underweight	Normal	Overweight or Obesity	*χ^2^*	*p* value
Age					110.367	**<0.001**
60–64	2,496 (39.5%)	111 (25.2%)	1,253 (38.4%)	1,132 (43.5%)		
65–69	1,817 (28.8%)	131 (29.8%)	911 (27.9%)	775 (29.8%)		
70–74	1,125 (17.8%)	86 (19.5%)	610 (18.7%)	429 (16.5%)		
75~	873 (13.8%)	112 (25.5%)	492 (15.1%)	269 (10.3%)		
Gender					76.577	**<0.001**
Male	3,072 (48.7%)	225 (51.1%)	1,749 (53.6%)	1,098 (42.1%)		
Female	3,239 (51.3%)	215 (48.9%)	1,517 (46.4%)	1,507 (57.9%)		
Education					22.616	**<0.001**
Illiterate	3,570 (56.6%)	270 (61.4%)	1,893 (58.0%)	1,407 (54.0%)		
Primary school	1,448 (22.9%)	98 (22.3%)	755 (23.1%)	595 (22.8%)		
Above middle school	1,293 (20.5%)	72 (16.4%)	618 (18.9%)	603 (23.1%)		
Marital status					10.703	**0.005**
Single, widow, divorced, and never married	5,148 (81.6%)	347 (78.9%)	2,628 (80.5%)	2,173 (83.4%)		
Married	1,163 (18.4%)	93 (21.1%)	638 (19.5%)	432 (16.6%)		
Type of residence					138.794	**<0.001**
Urban	1,509 (23.9%)	50 (11.4%)	651 (19.9%)	808 (31.0%)		
Rural	4,802 (76.1%)	390 (88.6%)	2,615 (80.1%)	1,797 (69.0%)		
Smoking status					170.863	**<0.001**
Never smoking	3,576 (56.7%)	208 (47.3%)	1,705 (52.2%)	1,663 (63.8%)		
Current smoking	1,744 (27.6%)	167 (38.0%)	1,082 (33.1%)	495 (19.0%)		
Former smoking	991 (15.7%)	65 (14.8%)	479 (14.7%)	447 (17.2%)		
Drinking status					38.489	**<0.001**
Never drinking	4,210 (66.7%)	298 (67.7%)	2,070 (63.4%)	1,842 (70.7%)		
Drink more than once a month	1,626 (25.8%)	116 (26.4%)	934 (28.6%)	576 (22.1%)		
Drink but less than once a month	475 (7.5%)	26 (5.9%)	262 (8.0%)	187 (7.2%)		
Eyesight					3.481	0.175
Good	972 (15.4%)	62 (14.1%)	483 (14.8%)	427 (16.4%)		
Poor	5,339 (84.6%)	378 (85.9%)	2,783 (85.2%)	2,178 (83.6%)		
Hearing					8.310	**0.016**
Good	1,838 (29.1%)	108 (24.5%)	930 (28.5%)	800 (30.7%)		
Poor	4,473 (70.9%)	332 (75.5%)	2,336 (71.5%)	1,805 (69.3%)		
Number of diseases					48.387	**<0.001**
0	1,714 (27.2%)	119 (27.0%)	986 (30.2%)	609 (23.4%)		
1	1,712 (27.1%)	119 (27.0%)	909 (27.8%)	684 (26.3%)		
≥2	2,885 (45.7%)	202 (45.9%)	1,371 (42.0%)	1,312 (50.4%)		
Social participation					30.507	**<0.001**
Yes	3,272 (51.8%)	210 (47.7%)	1,604 (49.1%)	1,458 (56.0%)		
No	3,039 (48.2%)	230 (52.3%)	1,662 (50.9%)	1,147 (44.0%)		
Activity of daily living					9.983	**0.007**
Normal	3,576 (56.7%)	218 (49.5%)	1,877 (57.5%)	1,481 (56.9%)		
Impaired	2,735 (43.3%)	222 (50.5%)	1,389 (42.5%)	1,124 (43.1%)		
Depression					27.305	**<0.001**
No	4,107 (65.1%)	246 (55.9%)	2,091 (64.0%)	1,770 (67.9%)		
Yes	2,204 (34.9%)	194 (44.1%)	1,175 (36.0%)	835 (32.1%)		
Sleep duration					7.159	0.128
<6 h	2,099 (33.3%)	154 (35.0%)	1,110 (34.0%)	835 (32.1%)		
6 ~ 8 h	3,592 (56.9%)	234 (53.2%)	1,832 (56.1%)	1,526 (58.6%)		
>8 h	620 (9.8%)	52 (11.8%)	324 (9.9%)	244 (9.4%)		
Average household income					10.483	**0.005**
Low	4,408 (69.8%)	2,313 (70.8%)	327 (74.3%)	1,768 (67.9%)		
High	1,903 (30.2%)	953 (29.2%)	113 (25.7%)	837 (32.1%)		
Occupation					95.134	**<0.001**
Unemployed	2,959 (46.9%)	1,372 (42.0%)	210 (47.7%)	1,377 (52.9%)		
Managers and professionals	572 (9.1%)	283 (8.7%)	26 (5.9%)	263 (10.1%)		
Agriculture and manual workers	2,780 (44.1%)	1,611 (49.3%)	204 (46.4%)	965 (37.0%)		

Bold values for *p* < 0.05.

### 3.2. Cognitive function of the study participants

Table 2 shows the characteristics of older participants. Of the 6,311 participants, 34.2% exhibited cognitive impairment. The difference in median scores on the MMSE scale between the cognitive impairment group and the normal cognitive group was statistically significant (16 vs. 24, *p* < 0.001). Additionally, the group afflicted with cognitive impairment exhibited a relatively lower BMI compared to the group with normal cognitive (22.72 kg/m^2^ vs. 23.44 kg/m^2^, *p* < 0.001). There were significant differences in BMI categories were also observed between the groups. Furthermore, the participants of the cognitive impairment group were older, more likely to be female, less educated, had worse hearing status, were less likely to participate in social activities, and were more likely to have a limited ability to perform ADL and depressive symptoms.

**Table 2:** Characteristics of participants with normal cognition and those with cognitive impairment (*n* = 6,311).

Characteristics	Classes	Normal cognition, *n* (%)	Cognitive impairment, *n* (%)	*χ^2^*	*p* value
No. of participants		4,153 (65.8%)	2,158 (34.2%)	-	**-**
Age				148.406	**<0.001**
	60–64	1,725 (41.5%)	771 (35.7%)		
	65–69	1,302 (31.4%)	515 (23.9%)		
	70–74	691 (16.6%)	434 (20.1%)		
	75~	435 (10.5%)	438 (20.3%)		
Gender				88.754	**<0.001**
	Male	2,199 (52.9%)	873 (40.5%)		
	Female	1,954 (47.1%)	1,285 (59.5%)		
Education				122.254	**<0.001**
	Illiterate	2,176 (52.4%)	1,394 (64.6%)		
	Primary school	1,119 (26.9%)	329 (15.2%)		
	Above middle school	858 (20.7%)	435 (20.2%)		
Marital status				79.559	**<0.001**
	Single, widow, divorced, and never married	3,518 (84.7%)	1,630 (75.5%)		
	Married	635 (15.3%)	528 (24.5%)		
Type of residence				122.622	**<0.001**
	Urban	1,171 (28.2%)	338 (15.7%)		
	Rural	2,982 (71.8%)	1,820 (84.3%)		
Body mass index				59.112	**<0.001**
	<18.5	226 (5.4%)	214 (9.9%)		
	18.5 ~ 23.9	2,114 (50.9%)	1,152 (53.4%)		
	≥24.0	1,813 (43.7%)	792 (36.7%)		
Smoking status				36.790	**<0.001**
	Never smoking	2,260 (54.4%)	1,316 (61.0%)		
	Current smoking	1,166 (28.1%)	578 (26.8%)		
	Former smoking	727 (17.5%)	264 (12.2%)		
Drinking status				8.859	**0.012**
	Never drinking	2,725 (65.6%)	1,485 (68.8%)		
	Drink more than once a month	1,119 (26.9%)	507 (23.5%)		
	Drink but less than once a month	309 (7.4%)	166 (7.7%)		
Eyesight				0.116	0.733
	Good	635 (15.3%)	337 (15.6%)		
	Poor	3,518 (84.7%)	1,821 (84.4%)		
Hearing				8.697	**0.003**
	Good	1,260 (30.3%)	578 (26.8%)		
	Poor	2,893 (69.7%)	1,580 (73.2%)		
Number of diseases				3.756	0.153
	0	1,156 (27.8%)	558 (25.9%)		
	1	1,101 (26.5%)	611 (28.3%)		
	≥2	1,896 (45.7%)	989 (45.8%)		
Social participation				35.278	**<0.001**
	Yes	2,265 (54.4%)	1,007 (46.7%)		
	No	1,888 (45.5%)	1,151 (53.3%)		
Activity of daily living				176.069	**<0.001**
	Normal	2,601 (62.6%)	975 (45.2%)		
	Impaired	1,552 (37.4%)	1,183 (54.8%)		
Depression				82.685	**<0.001**
	No	2,866 (69.0%)	1,241 (57.5%)		
	Yes	1,287 (31.0%)	917 (42.5%)		
Sleep duration				48.696	**<0.001**
	<6 h	1,307 (31.5%)	792 (36.7%)		
	6 ~ 8 h	2,488 (59.9%)	1,104 (51.2%)		
	>8 h	358 (8.6%)	262 (12.1%)		
Average household income				100.898	**<0.001**
	Low	2,727 (65.7%)	1,681 (77.9%)		
	High	1,426 (34.3%)	477 (22.1%)		
Occupation				27.468	**<0.001**
	Unemployed	1900 (45.8%)	1,059 (49.1%)		
	Managers and professionals	432 (10.4%)	140 (6.5%)		
	Agriculture and manual workers	1821 (43.8%)	959 (44.4%)		

Bold values for *p* < 0.05.

### 3.3. Association between BMI and cognitive status

The association between cognitive status and each baseline BMI category is shown in Table 3. Specifically, underweight individuals exhibited a considerably increased likelihood of cognitive impairment as compared to those in the normal baseline BMI group (OR = 1.738; 95% CI: 1.422–2.123). As shown in Table 3, this relationship between them still remained notable even after adjusting for age, gender, education, residence area, marital status, average household income, occupation, smoking status, alcohol consumption, hearing status, participation in social activities, nighttime sleep duration, ADL limitations, and depression (OR = 1.473; 95% CI: 1.189–1.823). Conversely, the risk of cognitive impairment in individuals with BMI in the overweight and obese categories was similar to that observed in individuals with a normal BMI.

**Table 3:** Results of logistic regression analyses of the association between BMI and cognition impairment.

Group	Model 1	Model 2	Model 3
Underweight (BMI<18.5 kg/m^2^)	**1.738 (1.422–2.123)**	**1.616 (1.315–1.984)**	**1.473 (1.189–1.823)**
Normal (18.5 ≤ BMI < 24.0 kg/m^2^)	1.000 (reference)	1.000 (reference)	1.000 (reference)
Overweight or obesity (BMI ≥ 24.0 kg/m^2^)	**0.802 (0.718–0.895)**	**0.783 (0.699–0.877)**	**0.874 (0.776–0.985)**

Model 1 was not adjusted for confounders. Model 2 was adjusted for age and gender. Model 3 was adjusted for age, gender, marital status, area of residence, education, average household income, occupation, smoking status, alcohol consumption, hearing status, ability to perform ADL, nighttime sleep duration, depressive symptoms, and social participation status. Bold values for *p* < 0.05.

### 3.4. Gender-based subgroup analysis

According to the gender-based subgroup analysis, older men and women with low weight exhibited a 52.2% (OR = 1.522; 95% CI: 1.123–2.062) and 42.3% (OR = 1.423; 95% CI: 1.048–1.932) higher risk of cognitive impairment, respectively, as compared to older adults with the normal baseline BMI. In contrast, overweight or obese older women showed a significantly decreased risk of cognitive impairment by 15.7% (OR = 0.843; 95% CI: 0.720–0.987); however, this relationship was not recognized in older men. The details are provided in Table 4.

**Table 4:** Subgroup analysis of the relationship between BMI and cognitive impairment according to gender.

Group	Model 1	Model 2	Model 3
Male			
Underweight (BMI<18.5 kg/m^2^)	**1.698 (1.277–2.256)**	**1.601 (1.199–2.138)**	**1.522 (1.123–2.062)**
Normal (18.5 ≤ BMI < 24.0 kg/m^2^)	1.000 (reference)	1.000 (reference)	1.000 (reference)
Overweight or obesity (BMI ≥ 24.0 kg/m^2^)	**0.774 (0.652–0.919)**	**0.807 (0.679–0.961)**	0.915 (0.759–1.104)
Female			
Underweight (BMI<18.5 kg/m^2^)	**1.769 (1.326–2.359)**	**1.652 (1.231–2.216)**	**1.423 (1.048–1.932)**
Normal (18.5 ≤ BMI < 24.0 kg/m^2^)	1.000 (reference)	1.000 (reference)	1.000 (reference)
Overweight or obesity (BMI ≥ 24.0 kg/m^2^)	**0.735 (0.634–0.851)**	**0.766 (0.659–0.889)**	**0.843 (0.720–0.987)**

Model 1 was not adjusted for confounders. Model 2 was adjusted for age and gender. Model 3 was adjusted for age, gender, marital status, area of residence, education, average household income, occupation, smoking status, alcohol consumption, hearing status, ability to perform ADL, nighttime sleep duration, depressive symptoms, and social participation status. Bold values for *p* < 0.05.

### 3.5. Dose–response relationships

**Figure 2:**
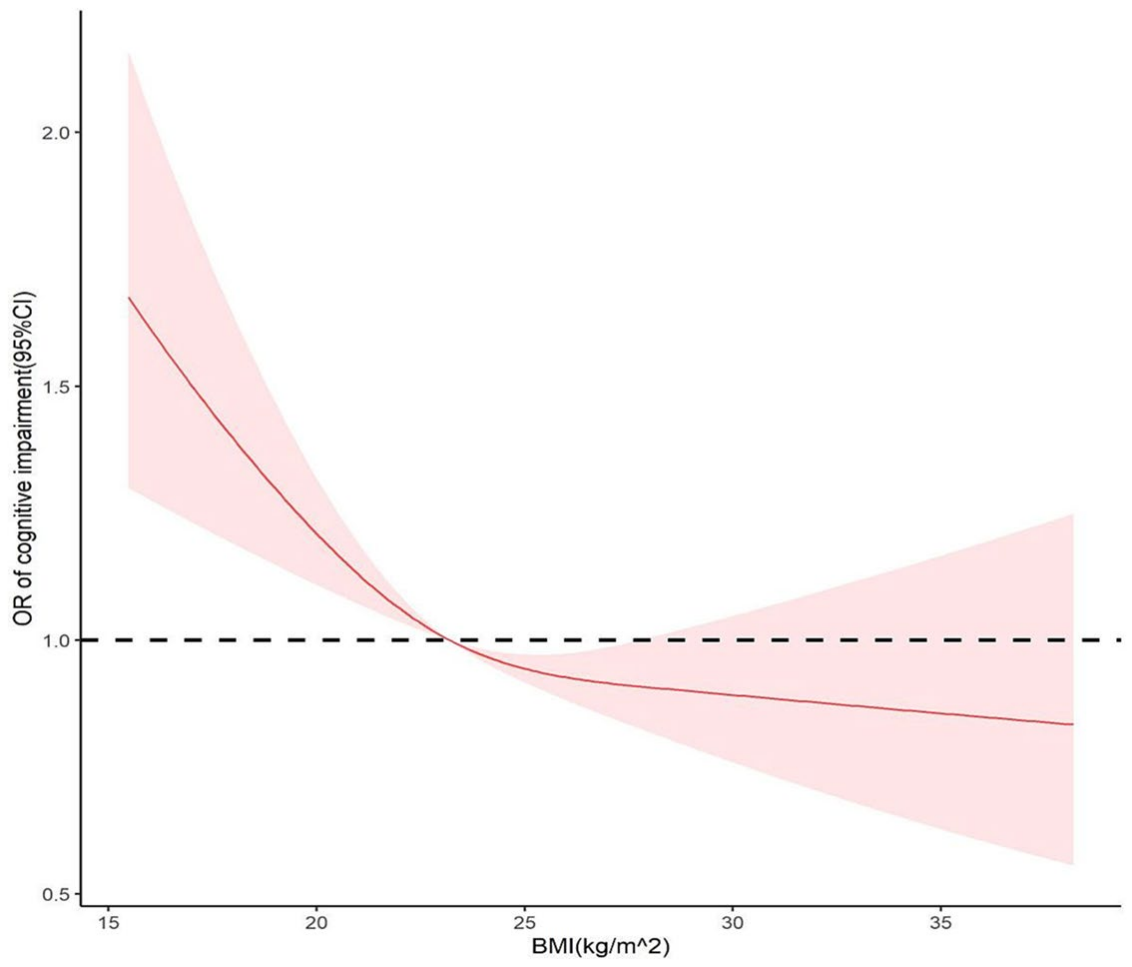
Restricted cubic splines for the association of baseline BMI with cognitive impairment.

To determine the association between BMI and cognitive impairment, a restricted cubic spline model was used to create dose–response curves, and BMI was considered a continuous variable. According to the Bare Pool Information Criterion, the 3-node model showed the smallest Akaike information criterion (AIC) value (7453.675); hence, the number of model nodes was chosen to be 3, as shown in Figure 2. After adjusting for confounders, the relationship between BMI and risk of cognitive impairment exhibited an L-shaped curve (*P* non-linearity <0.05). The results showed that the OR was higher at lower BMI levels, and as BMI approached 23.2 kg/m^2^, the OR was 1. Subsequently, there was a significant protective effect on cognitive function when BMI increased from 23.2 to 27.8 kg/m^2^.

## 4. Discussion

This nationwide prospective cohort study found a significant relationship between BMI and cognitive impairment in Chinese older adults. The results of the present study recommend that BMI could act as an important screening tool for the early identification of cognitive impairment in older adults. Even after adjusting for demographic and other significant factors, the analysis of the study data revealed that underweight older participants had a notable increased risk of cognitive impairment. Gender differences were also noted in the correlation between BMI and cognitive impairment. Older women who were overweight or obese were at a reduced risk of developing cognitive impairment; however, no such association was found in older men. The dose–response curve constructed using BMI as a continuous variable in the restricted cubic spline model revealed that the baseline BMI had a nonlinear relationship with the risk of cognitive impairment, with the ORs decreasing as BMI increased.

According to the results of study, the prevalence of cognitive impairment in Chinese older individuals was approximately 34.2%, with higher rates in women (39.7%) than in men (28.4%). The incidence of cognitive impairment in this study was higher than that in Shanghai (15.2%) (27), India (13.7%) (28), and Mexico (16.0%) (29). The reason for this higher incidence could be related to the differences in demographics, screening tools, and diagnostic criteria between the present study and the above-mentioned studies. The findings also indicated that the prevalence of cognitive impairment was significantly higher in older women than in older men. Previous studies in other countries or regions have found that male older adults have higher cognitive function scores than female older adults and that female older adults tend to have more severe cognitive impairment (30, 31). This finding was supported by the results of our study. Possible explanations for these differences between men and women can be attributed to less access of women to higher education (32) and changes in their estrogen levels during menopause (33). Estrogen plays a role in promoting neuronal growth and survival, which contributes the self-recovery of neurological deficits. Estrogen also exerts a protective effect on areas related to cognitive function, such as the cerebral cortex and hippocampus (34). Previous studies suggest that the decline in estrogen levels during menopause could significantly contribute to the pathogenesis of dementia, thereby elevating the risk of cognitive impairment (35, 36). Therefore, it is imperative to closely monitor the cognitive function of older individuals and use early screening tests, particularly for female seniors.

This study found that older individuals with a low BMI were at a significantly greater risk of experiencing cognitive impairment. Several previous studies also reported similar results (37–39). BMI serves as a significant objective indicator to estimate the nutritional status of seniors, and it plays a crucial role in the development and progression of neurodegenerative diseases associated with cognitive decline (40). Low BMI levels may be associated with sarcopenia, a degenerative condition of skeletal musculature that is characterized by gradual loss of muscle mass, vigor, and function (41). Sarcopenia is an age-related common senile disorder and has been broadly viewed as a dangerous factor for cognitive impairment (42, 43). An alternative explanation is that individuals with lower BMI may face more impediments to their physical mobility (44), which in turn could increase the risk of cognitive impairment (45, 46). Thus, it is extraordinarily important to provide targeted support to older adults who experience weight loss in their later years of life and continuously monitor their cognitive function.

Several previous findings support the protective effect of overweight or obesity on cognitive performance found in this study (8, 47–49). A 9-year longitudinal cohort study indicated an adjusted hazard ratio (AHR) of 0.86 (95% CI: 0.75–0.99) for cognitive impairment for the overweight group as compared to that for the normal weight group (8). A meta-analysis revealed a significant protective effect of being overweight and obese in older adults against cognitive impairment and dementia (7). The “obesity paradox” phenomenon may explain the protective effect of increased body weight on cognitive function in older individuals (50). High serum leptin levels may be a possible mechanism for the profitable effects of obesity on cognitive function in old age. With an increase in BMI, serum leptin levels also increase, and approximately 95% of obese patients have higher serum leptin levels compared to those with normal weight. Moreover, the serum leptin level is inversely proportional to the severity of cognitive impairment (51), indicating that the higher the serum leptin level, the larger the brain volume and the milder the extent of brain atrophy. Increased levels of leptin in the bloodstream can increase brain volume and decrease brain atrophy as well as contribute to the formation of synapses and growth of axons associated with neuroprotection (52). Animal experiments have shown that intracerebral injection of low-dose leptin in AD rats improved their cognition (53). These results suggest that leptin therapy has the potential to serve as an enhancer of cognitive functions.

Previous studies have also shown that being overweight or obese can increase the risk of dementia in older individuals (54–56). The differences in findings may be attributed to the differences in research samples, duration of follow-up, cognitive function measurement, types of dementia, obesity standards, and adjustment of potential confounding factors. A meta-analysis found different thresholds of BMI for the risk of developing cognitive impairment and dementia subtypes (Alzheimer’s disease and vascular dementia) (7). Future studies should specifically examine the association of BMI with the risk of cognitive impairment and dementia subtypes. Some potential mechanisms that could explain the impairment of cognitive function by obesity in old age include vitamin D deficiency (57, 58), inflammatory response (59), insulin resistance (60, 61), and oxidative stress (62). Further studies are required to investigate the correlation between obesity and cognition in older adults and its underlying mechanisms, and to provide strong evidence for proactive and effective preventive and therapeutic measures.

Interestingly, in the gender subgroup analysis, only older women who were obese or overweight showed a significantly lower risk of developing cognitive impairment; however, no such association was found in men. It was similar to the findings of a 3-year prospective study in Korea (15). According to previous studies, obesity accelerates cognitive decline in men more than in women (63, 64). An American study revealed that higher BMI protected against cognitive decline over 3 years in women, but not in men (64). Another cross-sectional study of older individuals in rural areas of China found a gender-dependent association between BMI and cognitive impairment. Older men with a higher BMI and women with a lower BMI were more prone to cognitive impairment (14). However, other studies on the association between obesity and cognitive decline have not found a gender difference (56). Hence, additional further multicenter prospective cohort studies with high quality are required to clarify this issue.

Our study has a significant advantage as we use an extensive, nationally representative, meticulously designed, prospective cohort of Chinese adults. Moreover, to the best of our knowledge, this present study is the first to use data from this database to assess the relationship between BMI and cognitive impairment in the Chinese older adults aged 60 years and above. We thoroughly analyzed BMI as a continuous variable, making full use of the available data. This present study enriched the study of the relationship between sex differences and dose–response between BMI and cognitive impairment. However, this study also has some limitations. First, despite we adjusted for several traditional sociodemographic characteristics as well as health- and lifestyle-related factors, we were unable to adjust for other unmeasured confounders such as medical treatment, diet, and APOE4 genotype that may mystify the association between BMI and cognitive impairment. Second, some confounding factors were based on self-reported data, such as sleep duration, household income, and social participation status, which may lead to recall bias. Third, the relationship between BMI and various forms of cognitive impairment like vascular cognitive impairment, frontotemporal dementia, mild cognitive impairment, and Alzheimer’s disease was not investigated because of insufficient data in this study. Additional multicenter prospective cohort studies are needed to detect the relationship between them. Fourth, the high proportion of overweight or obese and illiterate individuals in our study may limit the generalizability of our findings to the remaining older population, and further validation is required to clarify this aspect. Fifth, BMI alone may not be enough to symbolize fat accumulation. According to some studies, central obesity [waist circumference (WC) and waist-to-height ratio] is a helpful indicator of obesity in the aging population (10, 65). However, WC was not used to evaluate obesity in the present study; therefore, this indicator should be included in future research. Finally, considering that older adults with cognitive impairment may have difficulty eating or eating disorders, which can also result in a low BMI. Future studies could explore their bidirectional relationship.

## 5. Conclusion

In this large prospective national cohort study of older adults aged 60 years and above, our study found that low BMI in old age was associated with an increased risk of cognitive impairment. In contrast, being overweight or obese had a significant protective effect on cognitive function in Chinese older adults, and this association was more prominent in women. Our study suggests that maintaining a BMI in the range of 23.2–27.8 kg/m^2^ may help to sustain cognitive function. The results of this study have significant implications for the prevention of cognitive impairment. However, further prospective high-quality multicenter cohort studies are required to validate the L-shaped curve of BMI and cognitive impairment and to explain the underlying pathophysiological mechanisms. In conclusion, the findings of this study may help prevent cognitive impairment in older adults and promote healthy aging.

## Data availability statement

Publicly available datasets were analyzed in this study. This data can be found at: https://opendata.pku.edu.cn.

## Ethics statement

The studies involving humans were approved by Institutional Review Board of Peking University (IRB00001052-11015). The studies were conducted in accordance with the local legislation and institutional requirements. The participants provided their written informed consent to participate in this study.

## Author contributions

WD: Conceptualization, Data curation, Methodology, Writing – original draft, Writing – review & editing. LK: Conceptualization, Data curation, Writing – original draft. XZ: Data curation, Methodology, Writing – review & editing. ML: Software, Writing – review & editing. MW: Writing – original draft. YC: Supervision, Writing – review & editing.

## References

[1] National Bureau of Statistics of China. The Bulletin of the Seventh National Population Census (No.5). (2021). Available at: http://www.stats.gov.cn/tjsj/zxfb/202105/t20210510_1817181.html/ (Accessed May 11, 2021).

[2] Jia L Du Y Chu L Zhang Z Li F Lyu D. Prevalence, risk factors, and management of dementia and mild cognitive impairment in adults aged 60 years or older in China: a cross-sectional study. Lancet Public Health. (2020) 5:e661–71. doi: 10.1016/s2468-2667(20)30185-7, PMID: 33271079

[3] Jia L Quan M Fu Y Zhao T Li Y Wei C. Dementia in China: epidemiology, clinical management, and research advances. Lancet Neurol. (2020) 19:81–92. doi: 10.1016/s1474-4422(19)30290-x 31494009

[4] 2021 Alzheimer's disease facts and figures. Alzheimers Dement. (2021) 17:327–406. doi: 10.1002/alz.12328 33756057

[5] GBD 2016 Dementia Collaborators Nichols E Szoeke CEI Vollset SE Kivimäki M Meretoja A. Global, regional, and national burden of Alzheimer's disease and other dementias, 1990-2016: a systematic analysis for the global burden of disease study 2016. Lancet Neurol. (2019) 18:88–106. doi: 10.1016/s1474-4422(18)30403-4, PMID: 30497964 PMC6291454

[6] Jiang M Zou Y Xin Q Cai Y Wang Y Qin X. Dose-response relationship between body mass index and risks of all-cause mortality and disability among the elderly: a systematic review and meta-analysis. Clin Nutr. (2019) 38:1511–23. doi: 10.1016/j.clnu.2018.07.021, PMID: 30082166

[7] Qu Y Hu HY Ou YN Shen XN Xu W Wang ZT. Association of body mass index with risk of cognitive impairment and dementia: a systematic review and meta-analysis of prospective studies. Neurosci Biobehav Rev. (2020) 115:189–98. doi: 10.1016/j.neubiorev.2020.05.012, PMID: 32479774

[8] Wu S Lv X Shen J Chen H Ma Y Jin X. Association between body mass index, its change and cognitive impairment among Chinese older adults: a community-based, 9-year prospective cohort study. Eur J Epidemiol. (2021) 36:1043–54. doi: 10.1007/s10654-021-00792-y, PMID: 34370136

[9] Xiang X An R. Body weight status and onset of cognitive impairment among U.S. middle-aged and older adults. Arch Gerontol Geriatr. (2015) 60:394–400. doi: 10.1016/j.archger.2015.02.008, PMID: 25747849

[10] Ren Z Li Y Li X Shi H Zhao H He M. Associations of body mass index, waist circumference and waist-to-height ratio with cognitive impairment among Chinese older adults: based on the CLHLS. J Affect Disord. (2021) 295:463–70. doi: 10.1016/j.jad.2021.08.093, PMID: 34507227

[11] Liang F Fu J Turner-McGrievy G Wang Y Qiu N Ding K. Association of Body Mass Index and Plant-Based Diet with cognitive impairment among older Chinese adults: a prospective, Nationwide cohort study. Nutrients. (2022) 14:3132. doi: 10.3390/nu14153132, PMID: 35956314 PMC9370436

[12] Manacharoen A Jayanama K Ruangritchankul S Vathesatogkit P Sritara P Warodomwichit D. Association of body mass index and dietary intake with mild cognitive impairment and dementia: a retrospective cohort study. BMC Geriatr. (2023) 23:3. doi: 10.1186/s12877-022-03700-5, PMID: 36597023 PMC9808972

[13] O'Donovan G Sarmiento OL Hessel P Muniz-Terrera G Duran-Aniotz C Ibanez A. Associations of body mass index and sarcopenia with screen-detected mild cognitive impairment in older adults in Colombia. Front Nutr. (2022) 9:9. doi: 10.3389/fnut.2022.1011967, PMID: 36330135 PMC9623159

[14] Yuan Y Li J Zhang N Fu P Jing Z Yu C. Body mass index and mild cognitive impairment among rural older adults in China: the moderating roles of gender and age. BMC Psychiatry. (2021) 21:54. doi: 10.1186/s12888-021-03059-8, PMID: 33485307 PMC7825154

[15] Noh HM Han J Kim YJ Jung JH Roh YK Song HJ. Sex differences in the relationship between cognitive impairment and overweight or obesity in late life: a 3-year prospective study. Medicine (Baltimore). (2019) 98:e14736. doi: 10.1097/md.0000000000014736 30817627 PMC6831333

[16] Han F Luo C Lv D Tian L Qu C. Risk factors affecting cognitive impairment of the elderly aged 65 and over: a cross-sectional study. Front Aging Neurosci. (2022) 14:903794. doi: 10.3389/fnagi.2022.903794, PMID: 35783132 PMC9243469

[17] Zhao Y John S Yang G. Data from: China health and retirement longitudinal study (2011 baseline) Peking University Open Research Data Platform (2015).

[18] Zhao Y Hu Y Smith JP Strauss J Yang G. Cohort profile: the China health and retirement longitudinal study (CHARLS). Int J Epidemiol. (2014) 43:61–8. doi: 10.1093/ije/dys203, PMID: 23243115 PMC3937970

[19] The Chinese Working Group on Obesity. Guidelines for prevention and control of overweight and obesity in China (excerpts). Acta Nutrimenta Sinica. (2004) 1:1–4.

[20] Beeri MS Tirosh A Lin HM Golan S Boccara E Sano M. Stability in BMI over time is associated with a better cognitive trajectory in older adults. Alzheimers Dement. (2022) 18:2131–9. doi: 10.1002/alz.12525, PMID: 35049119 PMC9296696

[21] Folstein MF Folstein SE McHugh PR. Mini-mental state: a practical method for grading the cognitive state of patients for the clinician. J Psychiatr Res. (1975) 12:189–98. doi: 10.1016/0022-3956(75)90026-6 1202204

[22] Katzman R Zhang MY Ouang Ya Q Wang ZY Liu WT Yu E. A Chinese version of the Mini-mental state examination; impact of illiteracy in a Shanghai dementia survey. J Clin Epidemiol. (1988) 41:971–8. doi: 10.1016/0895-4356(88)90034-0 3193141

[23] Wang Z Zhang M. The Chinese version of the mini-mental state examination (MMSE) application. Shanghai Arch Psychiatry. (1989) 7:4.

[24] Gao Y Wei S Gao F Gao L Dang L Shang S. Sleep disturbance is associated with higher plasma Aβ levels in cognitively Normal adults-a population-based cross-sectional study. Front Aging Neurosci. (2020) 12:615838. doi: 10.3389/fnagi.2020.615838, PMID: 33536896 PMC7848159

[25] Yang M Hao Q Luo L Ding X Wu H Zhang Y. Body mass index and disability in Chinese nonagenarians and centenarians. J Am Med Dir Assoc. (2014) 15:303.e1–6. doi: 10.1016/j.jamda.2013.10.011, PMID: 24287207

[26] MacNeil A Birk S Villeneuve PJ Jiang Y de Groh M Fuller-Thomson E. Incident and recurrent depression among adults aged 50 years and older during the COVID-19 pandemic: a longitudinal analysis of the Canadian longitudinal study on aging. Int J Environ Res Public Health. (2022) 19:15032. doi: 10.3390/ijerph192215032, PMID: 36429749 PMC9690838

[27] Liu Y Yu X Han P Chen X Wang F Lian X. Gender-specific prevalence and risk factors of mild cognitive impairment among older adults in Chongming, Shanghai, China. Front Aging Neurosci. (2022) 14:900523. doi: 10.3389/fnagi.2022.900523, PMID: 36118698 PMC9475287

[28] Muhammad T Meher T. Association of late-life depression with cognitive impairment: evidence from a cross-sectional study among older adults in India. BMC Geriatr. (2021) 21:364. doi: 10.1186/s12877-021-02314-7, PMID: 34130632 PMC8204463

[29] Salinas-Rodríguez A Palazuelos-González R Rivera-Almaraz A Manrique-Espinoza B. Longitudinal association of sarcopenia and mild cognitive impairment among older Mexican adults. J Cachexia Sarcopenia Muscle. (2021) 12:1848–59. doi: 10.1002/jcsm.12787, PMID: 34535964 PMC8718052

[30] Tahami Monfared AA Byrnes MJ White LA Zhang Q. Alzheimer's disease: epidemiology and clinical progression. Neurol Ther. (2022) 11:553–69. doi: 10.1007/s40120-022-00338-8, PMID: 35286590 PMC9095793

[31] Bai W Chen P Cai H Zhang Q Su Z Cheung T. Worldwide prevalence of mild cognitive impairment among community dwellers aged 50 years and older: a meta-analysis and systematic review of epidemiology studies. Age Ageing. (2022) 51:afac173. doi: 10.1093/ageing/afac173, PMID: 35977150

[32] Nebel RA Aggarwal NT Barnes LL Gallagher A Goldstein JM Kantarci K. Understanding the impact of sex and gender in Alzheimer's disease: a call to action. Alzheimers Dement. (2018) 14:1171–83. doi: 10.1016/j.jalz.2018.04.008, PMID: 29907423 PMC6400070

[33] Conde DM Verdade RC Valadares ALR Mella LFB Pedro AO Costa-Paiva L. Menopause and cognitive impairment: a narrative review of current knowledge. World J Psychiatr. (2021) 11:412–28. doi: 10.5498/wjp.v11.i8.412, PMID: 34513605 PMC8394691

[34] Cheng D Liang B Hao Y Zhou W. Estrogen receptor α gene polymorphisms and risk of Alzheimer’s disease: evidence from a meta-analysis. Clin Interv Aging. (2014) 9:1031–8. doi: 10.2147/CIA.S65921, PMID: 25061285 PMC4085310

[35] Fu C Hao W Shrestha N Virani SS Mishra SR Zhu D. Association of reproductive factors with dementia: a systematic review and dose-response meta-analyses of observational studies. EClinicalMedicine. (2022) 43:101236. doi: 10.1016/j.eclinm.2021.101236, PMID: 34977513 PMC8683685

[36] Sochocka M Karska J Pszczołowska M Ochnik M Fułek M Fułek K. Cognitive decline in early and premature menopause. Int J Mol Sci. (2023) 24:6566. doi: 10.3390/ijms24076566, PMID: 37047549 PMC10095144

[37] Wang F Zhao M Han Z Li D Zhang S Zhang Y. Association of body mass index with amnestic and non-amnestic mild cognitive impairment risk in elderly. BMC Psychiatry. (2017) 17:334. doi: 10.1186/s12888-017-1493-x, PMID: 28915800 PMC5603057

[38] Zhang JJ Li L Liu D Hu FF Cheng GR Xu L. Urban-rural disparities in the association between body mass index and cognitive impairment in older adults: a cross-sectional study in Central China. J Alzheimers Dis. (2021) 83:1741–52. doi: 10.3233/jad-210295, PMID: 34459393

[39] Borda MG Venegas-Sanabria LC Garcia-Cifuentes E Gomez RC Cano-Gutierrez CA Tovar-Rios DA. Body mass index, performance on activities of daily living and cognition: analysis in two different populations. BMC Geriatr. (2021) 21:177. doi: 10.1186/s12877-021-02127-8, PMID: 33711937 PMC7953600

[40] Gómez-Gómez ME Zapico SC. Frailty, cognitive decline, neurodegenerative diseases and nutrition interventions. Int J Mol Sci. (2019) 20:2842. doi: 10.3390/ijms20112842, PMID: 31212645 PMC6600148

[41] Cruz-Jentoft AJ Sayer AA. Sarcopenia. Lancet. (2019) 393:2636–46. doi: 10.1016/s0140-6736(19)31138-9 31171417

[42] Hu F Liu H Liu X Jia S Zhao W Zhou L. Nutritional status mediates the relationship between sarcopenia and cognitive impairment: findings from the WCHAT study. Aging Clin Exp Res. (2021) 33:3215–22. doi: 10.1007/s40520-021-01883-2, PMID: 34028708 PMC8141547

[43] Cabett CipolliGSanches YassudaMAprahamianI. Sarcopenia is associated with cognitive impairment in older adults: a systematic review and Meta-analysis. J Nutr Health Aging. (2019) 23:525–31. doi: 10.1007/s12603-019-1188-8, PMID: 31233073

[44] Nascimento MM Gouveia ÉR Gouveia BR Marques A Campos P García-Mayor J. The mediating role of physical activity and physical function in the association between body mass index and health-related quality of life: a population-based study with older adults. Int J Environ Res Public Health. (2022) 19:13718. doi: 10.3390/ijerph192113718, PMID: 36360598 PMC9656348

[45] Wei X Liu H Yang L Gao Z Kuang J Zhou K. Joint developmental trajectories and temporal precedence of physical function decline and cognitive deterioration: a longitudinal population-based study. Front Psychol. (2022) 13:933886. doi: 10.3389/fpsyg.2022.933886, PMID: 36312122 PMC9597508

[46] Xu W Sun FR Tan CC Tan L. Weight loss is a preclinical signal of cerebral amyloid deposition and could predict cognitive impairment in elderly adults. J Alzheimers Dis. (2020) 77:449–56. doi: 10.3233/jad-200524 32675417

[47] Qizilbash N Gregson J Johnson ME Pearce N Douglas I Wing K. BMI and risk of dementia in two million people over two decades: a retrospective cohort study. Lancet Diabetes Endocrinol. (2015) 3:431–6. doi: 10.1016/s2213-8587(15)00033-9 25866264

[48] Singh-Manoux A Dugravot A Shipley M Brunner EJ Elbaz A Sabia S. Obesity trajectories and risk of dementia: 28 years of follow-up in the Whitehall II study. Alzheimers Dement. (2018) 14:178–86. doi: 10.1016/j.jalz.2017.06.2637, PMID: 28943197 PMC5805839

[49] Allen AN Clarke R Shipley M Leon DA. Adiposity in middle and old age and risk of death from dementia: 40-year follow-up of 19,000 men in the Whitehall study. Age Ageing. (2019) 48:247–53. doi: 10.1093/ageing/afy182, PMID: 30624572

[50] Dye L Boyle NB Champ C Lawton C. The relationship between obesity and cognitive health and decline. Proc Nutr Soc. (2017) 76:443–54. doi: 10.1017/s0029665117002014 28889822

[51] Tian J Wang T Jia K Guo L Swerdlow RH Du H. Nonobese male patients with Alzheimer's disease are vulnerable to decrease in plasma leptin. J Alzheimers Dis. (2022) 88:1017–27. doi: 10.3233/jad-220447, PMID: 35723107 PMC9553411

[52] Li TC Li CI Liu CS Lin CH Yang SY Lin CC. Obesity marker trajectories and cognitive impairment in older adults: a 10-year follow-up in Taichung community health study for elders. BMC Psychiatry. (2022) 22:748. doi: 10.1186/s12888-022-04420-1, PMID: 36451123 PMC9710179

[53] Oomura Y Hori N Shiraishi T Fukunaga K Takeda H Tsuji M. Leptin facilitates learning and memory performance and enhances hippocampal CA1 long-term potentiation and CaMK II phosphorylation in rats. Peptides. (2006) 27:2738–49. doi: 10.1016/j.peptides.2006.07.001, PMID: 16914228

[54] Feinkohl I Lachmann G Brockhaus WR Borchers F Piper SK Ottens TH. Association of obesity, diabetes and hypertension with cognitive impairment in older age. Clin Epidemiol. (2018) 10:853–62. doi: 10.2147/clep.S164793, PMID: 30100759 PMC6064155

[55] WangHHaiSLiuYXCaoLLiuYLiuP. Associations between Sarcopenic obesity and cognitive impairment in elderly Chinese community-dwelling individuals. J Nutr Health Aging. (2019) 23:14–20. doi: 10.1007/s12603-018-1088-3, PMID: 30569063

[56] Espeland MA Carmichael O Yasar S Hugenschmidt C Hazzard W Hayden KM. Sex-related differences in the prevalence of cognitive impairment among overweight and obese adults with type 2 diabetes. Alzheimers Dement. (2018) 14:1184–92. doi: 10.1016/j.jalz.2018.05.015, PMID: 30201101 PMC6338071

[57] Shea MK Barger K Dawson-Hughes B Leurgans SE Fu X James BD. Brain vitamin D forms, cognitive decline, and neuropathology in community-dwelling older adults. Alzheimers Dement. (2022) 19:2389–96. doi: 10.1002/alz.12836, PMID: 36479814 PMC10244481

[58] Gómez-Oliva R Geribaldi-Doldán N Domínguez-García S Carrascal L Verástegui C Nunez-Abades P. Vitamin D deficiency as a potential risk factor for accelerated aging, impaired hippocampal neurogenesis and cognitive decline: a role for Wnt/β-catenin signaling. Aging (Albany NY). (2020) 12:13824–44. doi: 10.18632/aging.103510, PMID: 32554862 PMC7377904

[59] Rui W Xiao H Fan Y Ma Z Xiao M Li S. Systemic inflammasome activation and pyroptosis associate with the progression of amnestic mild cognitive impairment and Alzheimer's disease. J Neuroinflammation. (2021) 18:280. doi: 10.1186/s12974-021-02329-2, PMID: 34856990 PMC8638109

[60] Arnold SE Arvanitakis Z Macauley-Rambach SL Koenig AM Wang HY Ahima RS. Brain insulin resistance in type 2 diabetes and Alzheimer disease: concepts and conundrums. Nat Rev Neurol. (2018) 14:168–81. doi: 10.1038/nrneurol.2017.185, PMID: 29377010 PMC6098968

[61] Kellar D Craft S. Brain insulin resistance in Alzheimer's disease and related disorders: mechanisms and therapeutic approaches. Lancet Neurol. (2020) 19:758–66. doi: 10.1016/s1474-4422(20)30231-3, PMID: 32730766 PMC9661919

[62] Song T Song X Zhu C Patrick R Skurla M Santangelo I. Mitochondrial dysfunction, oxidative stress, neuroinflammation, and metabolic alterations in the progression of Alzheimer's disease: a meta-analysis of in vivo magnetic resonance spectroscopy studies. Ageing Res Rev. (2021) 72:101503. doi: 10.1016/j.arr.2021.101503, PMID: 34751136 PMC8662951

[63] Chen N Cao J Zhang W Chen Y Xu L. Gender differences in the correlation between body mass index and cognitive impairment among the community-dwelling oldest-old in China: a cross-sectional study. BMJ Open. (2022) 12:e065125. doi: 10.1136/bmjopen-2022-065125, PMID: 36418136 PMC9685246

[64] Rodríguez-Fernández JM Danies E Martínez-Ortega J Chen WC. Cognitive decline, body mass index, and waist circumference in community-dwelling elderly participants. J Geriatr Psychiatry Neurol. (2017) 30:67–76. doi: 10.1177/0891988716686832, PMID: 28077009

[65] Tang X Zhao W Lu M Zhang X Zhang P Xin Z. Relationship between central obesity and the incidence of cognitive impairment and dementia from cohort studies involving 5,060,687 participants. Neurosci Biobehav Rev. (2021) 130:301–13. doi: 10.1016/j.neubiorev.2021.08.028, PMID: 34464646

