# Supplementary material for: Association between body mass index and cognitive impairment in Chinese older adults
Source: Front Public Health. 2023 Oct 18;11:1255101. doi: 10.3389/fpubh.2023.1255101 (PMC10622794; doi:10.3389/fpubh.2023.1255101)
Supplement: Supplementary file 1 [file Table_1.DOCX]

Supplementary Material

Association between body mass index and cognitive impairment in Chinese elderly people

Wenshuo Dong, Lichao Kan, Xinyue Zhang, Mengli Li, Meijuan Wang, Yingjuan Cao^*^

*** Correspondence:** Yingjuan Cao: caoyj@sdu.edu.cn

# analysis code

#-----Analysis script for restricted cubic splines between bmi and cognitive impairment----------

# Load packages ---------------------------------------------------------------------------------

setwd("D:/papers/rcs")

library(tibble)

library(ggplot2)

library(rms)

library(foreign)

# Load and process data -------------------------------------------------------------------------

rcs <- read.spss("D:/papers/rcs/bmi.sav",to.data.frame = T)

# check data

dim(rcs)

summary (rcs)

# Set up the data environment for the subsequent programs

dd <- datadist(rcs)

options(datadist = "dd")

# Fitted model -------------------------------------------------------------------------------------

fit3 <- lrm(CI ~rcs(BMI,3)

+age+gender+education+residence+marriage+smoking+drinking+hearing

+activity+adl+depression+sleeptime+income+work,

data=rcs)

fit4 <- lrm(CI ~rcs(BMI,4)

+age+gender+education+residence+marriage+smoking+drinking+hearing

+activity+adl+depression+sleeptime+income+work,

data=rcs)

fit5 <- lrm(CI ~rcs(BMI,5)

+age+gender+education+residence+marriage+smoking+drinking+hearing

+activity+adl+depression+sleeptime+income+work,

data=rcs)

# Nonlinearity test --------------------------------------------------------------------------------

anova(fit3)

anova(fit4)

anova(fit5)

AIC(fit3)

AIC(fit4)

AIC(fit5)

fit3

OR3 <- Predict(fit3,BMI,fun=exp,ref.zero = TRUE)

# Output -------------------------------------------------------------------------------------------

ggplot()+

geom_line(data=OR3, aes(BMI,yhat),

linetype="solid",size=0.55,alpha = 0.7,colour="#ff0000")+

geom_ribbon(data=OR3,

aes(BMI,ymin = lower, ymax = upper),

alpha = 0.1,fill="#ff0000")+

theme_classic()+

geom_hline(yintercept=1, linetype=2,size=1)+

labs( x="BMI(kg/m^2)", y="OR of cognitive impairment(95%CI)")

ggsave(filename = "RCS.jpg",

width = 7,

height = 7,

units = "in",

dpi = 300)
